# Cardiac Remodeling: Endothelial Cells Have More to Say Than Just NO

**DOI:** 10.3389/fphys.2018.00382

**Published:** 2018-04-11

**Authors:** Vincent F. M. Segers, Dirk L. Brutsaert, Gilles W. De Keulenaer

**Affiliations:** ^1^Laboratory of Physiopharmacology, University of Antwerp, Antwerp, Belgium; ^2^Department of Cardiology, University Hospital Antwerp, Edegem, Belgium; ^3^Department of Cardiology, Middelheim Hospital, Antwerp, Belgium

**Keywords:** cardiac remodeling, endothelium, intercellular communication, proteins, heart failure

## Abstract

The heart is a highly structured organ consisting of different cell types, including myocytes, endothelial cells, fibroblasts, stem cells, and inflammatory cells. This pluricellularity provides the opportunity of intercellular communication within the organ, with subsequent optimization of its function. Intercellular cross-talk is indispensable during cardiac development, but also plays a substantial modulatory role in the normal and failing heart of adults. More specifically, factors secreted by cardiac microvascular endothelial cells modulate cardiac performance and either positively or negatively affect cardiac remodeling. The role of endothelium-derived small molecules and peptides—for instance NO or endothelin-1—has been extensively studied and is relatively well defined. However, endothelial cells also secrete numerous larger proteins. Information on the role of these proteins in the heart is scattered throughout the literature. In this review, we will link specific proteins that modulate cardiac contractility or cardiac remodeling to their expression by cardiac microvascular endothelial cells. The following proteins will be discussed: IL-6, periostin, tenascin-C, thrombospondin, follistatin-like 1, frizzled-related protein 3, IGF-1, CTGF, dickkopf-3, BMP-2 and−4, apelin, IL-1β, placental growth factor, LIF, WISP-1, midkine, and adrenomedullin. In the future, it is likely that some of these proteins can serve as markers of cardiac remodeling and that the concept of endothelial function and dysfunction might have to be redefined as we learn more about other factors secreted by ECs besides NO.

## Introduction

**Figure 1:**
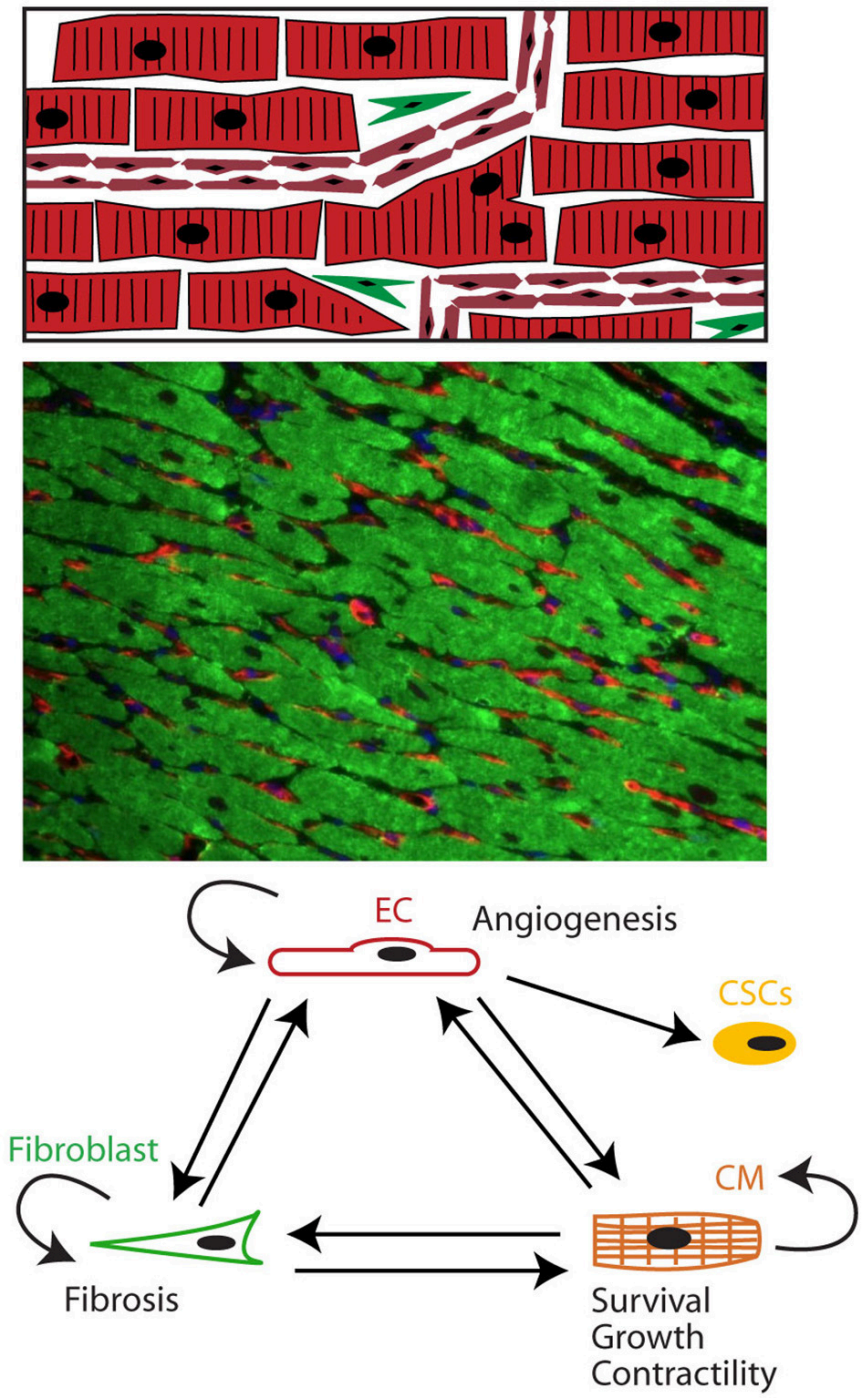
The heart as a pluricellular organ. **(Upper)** The heart is a highly organized pluricellular tissue consisting of myocytes (red, striated), capillary ECs (red, smaller elongated cells), and to a lesser extent fibroblasts (green spindle shaped) and stem cells. **(Middle)** Fluorescent staining of myocardial tissue with myocytes depicted in green and endothelial cells in red. Myocytes and endothelial cells are in close contact with each other. **(Lower)** Cells communicate through autocrine, juxtacrine and paracrine signals.

The heart is a muscular pump consisting of myocytes, endothelial cells (ECs), fibroblasts, stem cells, and inflammatory cells (Segers and Lee, 2008; Kamo et al., 2015). Cardiac tissue is a highly organized structure of cells and extracellular matrix with an intricate multidirectional communication between cells. All cells present in the myocardium secrete autocrine, juxtacrine, and paracrine factors that modulate function of neighboring cells (Figure 1). Intercellular communication plays crucial roles in cardiac development and normal cardiac function in the adult organism, but also in the pathophysiology of cardiac remodeling and heart failure development. In particular, factors secreted by cardiac microvascular ECs play a crucial role in normal cardiac function and during cardiac remodeling.

The role of endothelium-derived small molecules and peptides has been extensively studied and is relatively well defined. For instance, nitric oxide (NO) affects cardiac contractility by inducing an earlier onset of relaxation resulting in a longer diastole and favoring diastolic filling (Brutsaert, 2003; Balligand et al., 2009). Another example is endothelin-1, which has positive inotropic effects (Moravec et al., 1989) and induces a hypertrophic response in cardiomyocytes (Drawnel et al., 2013). However, ECs do not only secrete small molecules and peptides but also numerous proteins. Information on the role of these proteins in normal cardiac biology and cardiac remodeling is limited and scattered throughout the literature.

Another issue is that the cardiotrophic effects of certain secreted proteins are not always linked to the source of the proteins, which is in a number of instances the cardiac microvascular endothelium. In recent years, a number of excellent papers have been published describing cardioprotective effects of specific endogenous proteins (Oshima et al., 2008; Shimano et al., 2011; Frangogiannis, 2012; Zhang et al., 2014), without discussing their source. Moreover, signaling proteins in the heart are sometimes referred to as “matricellular proteins” (Frangogiannis, 2012), a term that ignores the origin of these proteins and suggests that they are a static part of the extracellular matrix. Cardiac microvascular ECs are the most abundant cell type—not in total volume but in total number—in adult myocardium (Pinto et al., 2015), are in direct contact with adjacent cardiomyocytes and fibroblasts, and actively secrete many proteins.

In this review, we will link specific proteins that modulate cardiac contractility or cardiac remodeling to their expression by cardiac microvascular ECs using publicly available expression libraries. In physiology, there are numerous feed-back and feed-forward mechanisms that are part of an intricate multidirectional communication network. Similar feed-back and feed-forward mechanisms are present in the communication between ECs, cardiomyocytes, and fibroblasts in the heart. For example, when ECs send a signal to cardiomyocytes, these will respond with a signal that enhances or attenuates the original signal. To limit the degree of complexity in this review, we will focus on signals secreted by microvascular ECs present in the myocardium and ignore signals from other cells. We will narrow the focus of this review further by discussing endothelial-derived proteins; many excellent reviews can be found on small molecules and peptides secreted by cardiac ECs (Brutsaert, 2003; Chatzizisis et al., 2007; Duncker and Bache, 2008; Kamo et al., 2015; Lim et al., 2015). The overall aim of the present review is to provide new insights in the role of microvascular endothelial cells in pathophysiology of cardiac remodeling beyond secretion of NO. Furthermore, we want to summarize evidence about either the protective or the adverse effect of endothelium-derived proteins, regarding to cardiac contractility, cardiac remodeling, and different cardiac diseases.

## Methodology

Inclusion of endothelial-derived proteins in this review was based on publicly available micro-array datasets in Geo Datasets (Table 1). Micro-array data were extracted from GSE45820 which contained mRNA expression levels of CD31 positive cardiac ECs isolated with flow cytometry cell sorting. These cardiac ECs were derived from mice with or without thoracic aorta constriction (TAC) (Moore-Morris et al., 2014). Relative gene expression between cardiac ECs from TAC mice was compared to wild type mice. We selected all genes encoding secreted proteins with at least a two-fold upregulation in mRNA expression and with a known function in adult cardiac physiology. The advantage of this strategy is that proteins were selected in a non-biased way. However, many secreted endothelial-derived proteins with important functions in cardiac biology will be missed using this strategy. For instance, neuregulin-1 upregulation in this micro-array database was less than two-fold, but its important roles in endothelial-cardiomyocyte communication have been well-established (Lemmens et al., 2006). Furthermore, one has to bear in mind that none of these proteins is produced exclusively by ECs. Proteins secreted by one specific cell type are rare in mammalian biology. Nevertheless, an endothelial source is an important physiological characteristic for a secreted protein, allowing specific regulatory features of synthesis and secretion and providing anatomical advantages for action. However, the endothelium is a fragile organ, easily disturbed during aging and environmental stimuli (Table 2). Therefore, an endothelial origin of a pathway may reveal an enhanced vulnerability of this pathway to certain (patho-)physiological conditions.

**Table 1:** Data sets used in this manuscript.

**Dataset**	**Description**	**Species**	**References**
GSE45820	Endothelial gene profiling following pressure overload	Mice	Moore-Morris et al., 2014
GDS1402	Various normal pure cell cultures	Human	
GDS2206	Dilated cardiomyopathy (human)	Human	Barth et al., 2006
GSE26887	Ischemic cardiomyopathy	Human	Greco et al., 2012
GDS3661	Hypertensive cardiomyopathy	Rats	Brooks et al., 2010
GDS1264	Hypertensive cardiomyopathy	Rats	Rysa et al., 2005
GDS3655	Ischemic cardiomyopathy	Mice	Lachtermacher et al., 2010
GDS2145	7 days post myocardial infarction	Rats	Andersson et al., 2006
GDS2424	Pacing induced heart failure	Dogs	Ojaimi et al., 2007
GDS2154	Inflammatory cardiomyopathy (parvovirus induced)	Human	Wittchen et al., 2007
GDS3228	TAC in apelin-KO mice	Mice	Kuba et al., 2007
GDS2773	Acute and chronic EC response to TNF-α	Mice	Rajashekhar et al., 2007
GDS1543	EC response to TNF-α	Human	
GDS1968	EC response to hypoxia and reoxygenation	Human	

**Table 2:** Relative expression of angiocrine proteins upon TNF-α or hypoxia in cell culture.

**Gene**	**Protein**	**GDS2773**	**GDS2773**	**GDS1543**	**GDS1968**	**GDS1968**	**GDS1968**	**GDS1968**	**GDS1968**
		**mouse EC**	**mouse EC**	**HMEC**	**HUVEC**	**HUVEC**	**HUVEC**	**HUVEC**	**HUVEC**
		**TNF acute**	**TNF chronic**	**TNF**	**1 h hypox**	**3 h reox**	**5 h reox**	**12 h reox**	**24 h reox**
Tnc	Tenascin C			4.8	2.1	2.3			
Thbs1	Thrombospondin 1	13.4			0.6			0.6	
Fstl1	Follistatin-like 1	2.1							
Ctgf	Connective tissue growth factor	4.5				0.4		0.7	
Ptgis	Prostaglandin I2 synthase				0.6			0.5	
Bmp2	Bone morphogenetic protein 2				0.7				
Apln	Apelin				0.6				
Thbs2	Thrombospondin 2				4.7	3.9	4.7	3	
Thbs3	Thrombospondin 3				1.6				
Il1b	Interleukin 1 beta			4.1					
Pgf	Placental growth factor				1.4				
Lif	leukemia inhibitory factor		3.2		1.5	2.5			
Tnxb	Tenascin XB								1.7
Wisp1	WNT1 inducible signaling pathway protein 1					0.6	0.5	0.5	
Mdk	Midkine				1.5				

*Relative expression of angiocrine proteins in ECs after acute or chronic stimulation with TNF-α, after 1 h ischemia, or after different time points of reoxygenation. All experiments are based on in vitro EC culture and all values are compared to control samples (non-stimulated or non-ischemic). Only statistically significant differences are shown*.

This review is based on comprehensive search of peer-reviewed literature on PubMed. Inclusion of references was based on relevance to the topic, quality of the manuscript and consistency with the literature. Search terms included the following: heart, cardiac, ECs, endothelium, cardiomyocytes, fibroblasts, stem cell, hypertrophy, cardiac fibrosis, heart failure, cardiomyopathy, lactate, oxygen, vasodilation, mechanotransduction, adenosine, and vasopressin.

Furthermore, we checked expression of these proteins in cardiac ECs and other ECs using other publically available expression data sets. We confirmed the expression of all proteins mentioned in this manuscript using publicly available datasets of various normal pure human cells including cardiac microvascular ECs and cardiac fibroblasts (GDS1402, Table 3). We also checked expression of these genes in samples from human myocardial biopsies of patients with dilated cardiomyopathy (GDS2206), samples from hypertrophic hearts induced by exercise in rats (GDS654), and samples from hypertrophic hearts from various induction models (GDS598).

**Table 3:** Relative expression of angiocrine proteins in models of cardiac overload or compared to other cell types.

**Gene**	**Protein**	**A**	**B**	**C**	**D**	**E**	**F**	**G**	**H**	**I**	**J**	**K**	**L**
		**GSE45820**	**GDS1402**	**GDS1402**	**GDS3661**	**GDS1264**	**GDS3288**	**GDS3655**	**GDS2145**	**GDS2542**	**GDS2206**	**GDS2424**	**GDS2154**
		**Mouse**	**Human**	**Human**	**Rat**	**Rat**	**Mouse**	**Mouse**	**Rat**	**Mouse**	**Human**	**Dog**	**Human**
		**TAC**	**vs. fibro**	**vs. SMC**	**AHT HF**	**AHT HF**	**Apelin KO**	**ICMP**	**ICMP**	**Obesity HF**	**DCMP**	**Pacing HF**	**Myocarditis**
Il6	Interleukin 6	93.4	1.5		0.5	0.6							
Postn	Periostin	46.7		0.2		11.5	6.3	3.4				3.7	
Tnc	Tenascin C	24.5			0.2								
Thbs1	Thrombospondin 1	24.1	8.7	6.1	0.4							3.8	3.1
Fstl1	Follistatin-like 1	12.6	1.9			2.0	2.6				0.9		0.6
Frzb	Frizzled-related protein 3	9.7	0.3	0.5	4.7								2.3
Thbs4	Thrombospondin 4	9.5				31.2	3.6			0.5	1.5		
Igf1	Insulin-like growth factor 1	9.1									1.1		
Ctgf	Connective tissue growth factor	8.7	1.9	1.3			3.1				1.4		
Ptgis	Prostaglandin I2 synthase	7.4	0.07	0.2		2.1				1.6			
Dkk3	Dickkopf homolog 3	6.5			1.5						1.5		2.0
Bmp2	Bone morphogenetic protein 2	5.3											
Apln	Apelin	4.7	6.1	7.4									0.7
Thbs2	Thrombospondin 2	4.1	0.4	0.4				1.9			1.1	7.8	
Thbs3	Thrombospondin 3	3.6											
Il1b	Interleukin 1 beta	3.5	0.06	0.06						0.4			
Pgf	Placental growth factor	3.4	13.2	16.8	0.8								
Ace	Angiotensin I converting enzyme 1	3.2	1.4	1.6		1.6	2.2	2.2	1.8				
Lif	Leukemia inhibitory factor	3	0.8	0.8	0.7								
Bmp4	Bone morphogenetic protein 4	2.7	9.4	3.5	1.6				0.6				
Tnxb	Tenascin XB	2.7	0.8										0.5
Wisp1	WNT1 inducible signaling pathway protein 1	2.6	0.8	0.4							0.9		1.9
Mdk	Midkine	2.5	2.3	1.8							0.9		0.5
Adm	Adrenomedullin	2.1	0.3	0.4							1.2	2.6	

*(A) Relative expression of angiocrine proteins in cardiac microvascular ECs of mice after thoracic aortic constriction compared to sham operated mice; microarray data of flow cytometry sorted cardiac microvascular ECs (n = 1) (Moore-Morris et al., 2014). (B) Relative expression in various EC cultures (n = 14) compared to various fibroblast cultures (n = 7). (C) Relative expression in various EC cultures (n = 14) compared to various smooth muscle cell cultures (n = 26). (D,E) Relative expression in myocardium of rats with hypertensive cardiomyopathy compared to control animals. (F) Relative expression in myocardium of apelin-KO mice compared to control. (G,H) Relative expression in remote myocardium after myocardial infarction. (I) Relative expression in obesity-induced heart failure in mice compared to controls. (J) Relative expression in myocardial biopsy samples of patients with dilated cardiomyopathy compared to normal cardiac samples (Barth et al., 2006). (K) Relative expression in myocardium of pacing induced heart failure in dogs compared to control samples. (L) Relative expression in myocardial biopsy samples of patients with myocarditis compared to normal cardiac samples. Column B–L: only significant differences are shown*.

## Cardiac microvascular endothelial cells

**Figure 2:**
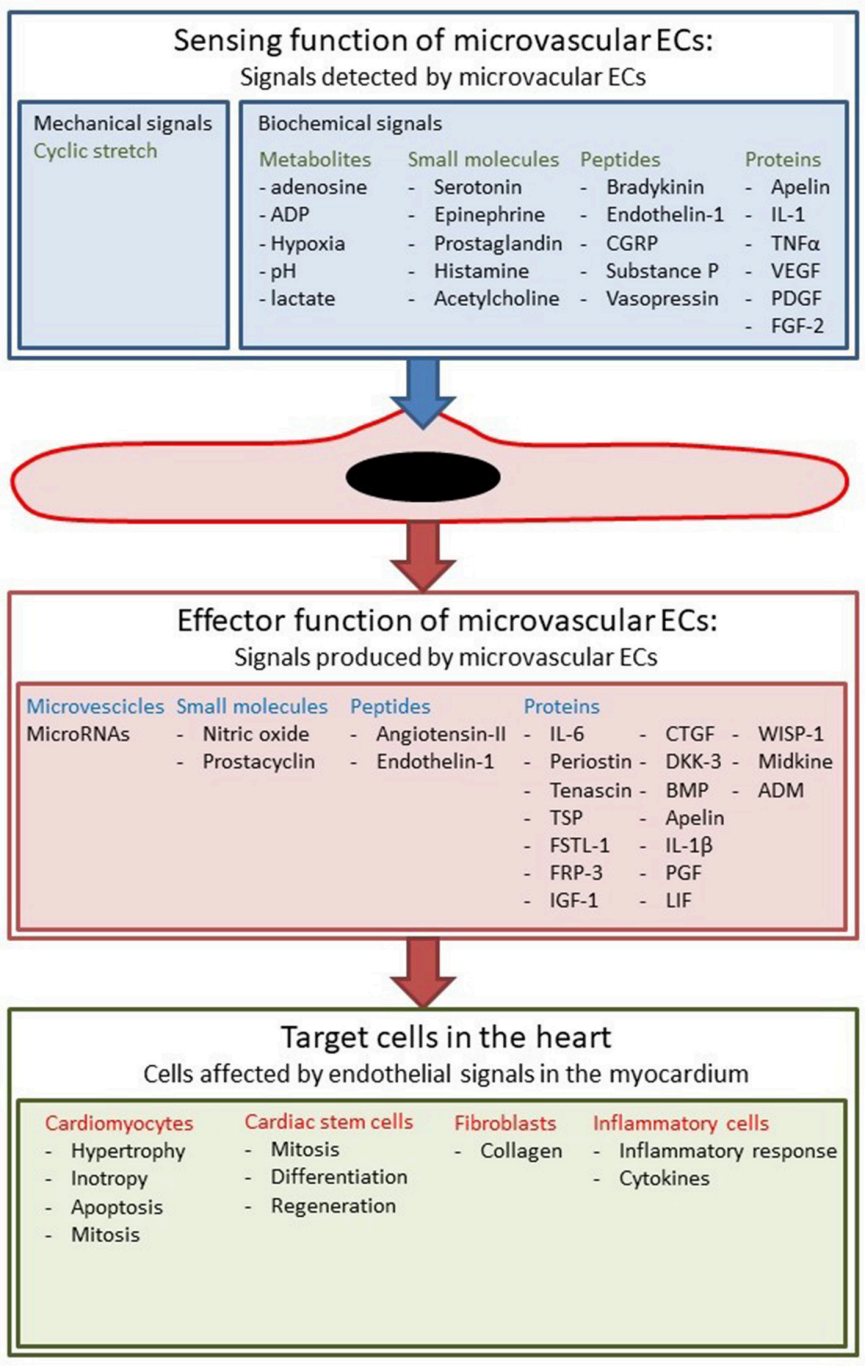
Sensing and effector function of cardiac ECs. ECs sense different biochemical and mechanical stimuli and communicate with other cell types in the myocardium.

Cardiac muscle is a tissue with high metabolic needs and therefore receives blood supply from a dense vascular and capillary network. Capillary density in the myocardium is around 3,000–4,000/mm^2^, which is substantially higher than in skeletal muscle where it is around 500–2,000/mm^2^ (Duncker and Bache, 2008). Microvascular ECs lining these capillaries not only serve as a barrier between blood and the myocardial tissue, but also communicate with adjacent cardiomyocytes by exchanging small molecules, peptides, proteins, microvesicles, and microRNAs (Figure 2) (Brutsaert, 2003). These secreted angiocrine substances constitute the endothelial *effector function* of the myocardium. Conceptually, one could discriminate the effector functions based on the target cell type, but alternatively one could also discriminate based on target processes, e.g., hypertrophy or fibrosis. The effector function of ECs has been first described almost 30 years ago, when it was shown that vascular ECs produce NO which induces relaxation of underlying smooth muscle cells (Palmer et al., 1987). Subsequently it has been shown that NO produced by endocardial endothelium also modulates contractility of cardiomyocytes (Brutsaert, 2003). Later, it has been shown that ECs communicate with cardiomyocytes by other signal molecules including prostaglandins and short peptides like endothelin (Brutsaert, 2003; Kamo et al., 2015). In recent years it has also been shown that proteins can modulate cardiac contractility (Lemmens et al., 2004) and have protective effects on cardiac remodeling (Liu et al., 2006): the best characterized example is neuregulin-1 (Vermeulen et al., 2016, 2017).

Besides this effector function, ECs also have a *sensing function* to detect changes in hemodynamic, chemical, neurohormonal, and mechanical stimuli (Figure 2). The best known example of this sensing function is the secretion of vasodilatory substances such as NO in response to changes in shear stress (Chatzizisis et al., 2007; Duncker and Bache, 2008; Davies, 2009). However, shear stress is only important in arteries and larger arterioles, because flow rates in the microcirculation are much lower (Boulpaep, 2009). Nevertheless, ECs in specific microcirculations such as the heart or skeletal muscle are subjected to mechanical stress such as cyclical stretching and compression, and load-dependent strain. Furthermore, all ECs have receptors for metabolites, neurohormonal factors, cytokines, and growth factors; they harbor these receptors not only to regulate their own cellular physiology, but also to transduce signals to neighboring cells, for instance underlying cardiomyocytes. An interesting example is the responsiveness of ECs to estrogens by secreting more NO, a phenomenon that could explain some of the gender differences in many cardiovascular diseases (Gavin et al., 2009).

## Cardiac endothelial cells secrete small molecules that modulate cardiac contractility and cardiac remodeling

ECs have many effector functions that occur in different organs such as regulation of coagulation or inflammatory cell infiltration, but they have also effector functions that are specific to certain tissues. ECs located in epicardial coronary arteries are a small minority of all ECs in the heart, but their role in modulating vascular smooth muscle function is extensively studied (Duncker and Bache, 2008). In this review we focus on the effector function of the microvascular ECs in the heart.

**Figure 3:**
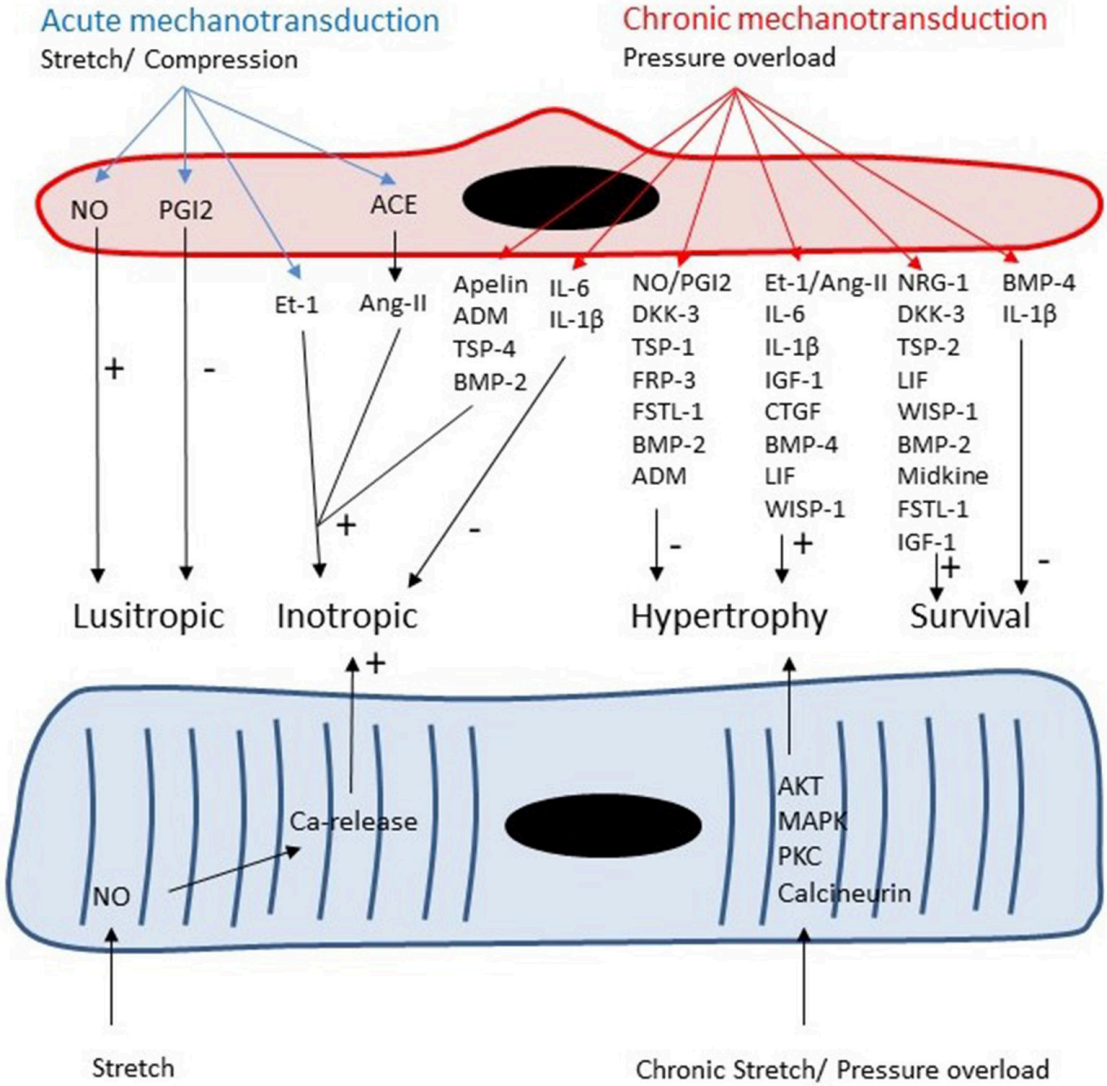
Both cardiomyocytes and microvascular ECs are responsive to acute and chronic changes in loading conditions. Autocrine and paracrine signaling leads to acute changes in lusitropy and inotropy of cardiomyocytes and to chronic changes in cardiomyocyte growth and survival.

The vast majority of ECs in the heart are located in the microcirculation. These ECs produce paracrine factors, which can modulate cardiomyocyte contractility, growth and survival (Figure 3). Similar to ECs in coronary arteries, these paracrine factors include NO, prostacyclin, Ang-II, and ET-1 (Brutsaert, 2003). Detailed discussion of the paracrine effects of *NO* is outside the scope of this review (Brutsaert, 2003; Balligand et al., 2009). In small concentrations, NO has positive inotropic effects, whereas in higher concentrations it has negative inotropic effects (Mohan et al., 1996; Brutsaert, 2003; Balligand et al., 2009). The most reproducible effect of NO on cardiac contractility, however, is an earlier onset of relaxation (positive lusitropy) resulting in a longer diastole and favoring diastolic filling and coronary perfusion (Brutsaert, 2003; Balligand et al., 2009). In the long run, production of NO by endothelial NOS has antihypertrophic effects in models of cardiac hypertrophy (Palmer et al., 1987; Massion and Balligand, 2007). Paulus et al. recently proposed a novel paradigm for pathophysiology of heart failure with preserved ejection fraction (HFpEF). In this paradigm, a co-morbidity-induced dysfunction of cardiac microvascular endothelium plays a central role in development of cardiomyocyte hypertrophy and stiffness (Paulus and Tschope, 2013). Microvascular endothelial dysfunction leads to decreased NO production, decreased cGMP content and protein kinase G (PKG) activity in adjacent cardiomyocytes which results in development of hypertrophy and increased cardiomyocyte stiffness (Paulus and Tschope, 2013).

The effects of *prostacyclin* on cardiac contractility range from a positive to a negative inotropic effect (Brutsaert, 2003). The main effect of prostacyclin on contractility is a delayed onset of relaxation and this effect opposes the action of NO (Brutsaert, 2003). The role of prostacyclin in cardiac remodeling is less well defined, but there is evidence that prostacyclin has anti-hypertrophic effects (Ritchie et al., 2004) and that the hypertrophic response is exaggerated in prostacyclin-receptor knockout mice (Hara et al., 2005; Harding and Murray, 2011). For the effects of other prostaglandins on cardiac remodeling, we refer the reader to ref (Harding and Murray, 2011). In the dataset used in this manuscript to select endothelium-derived proteins, prostaglandin I2 synthase mRNA is upregulated 7.4-fold in ECs derived from left ventricle of mice after aortic banding (Table 3).

Locally produced *Ang-II* is important in normal cardiac function with the most consistent effect being positive inotropy (Freer et al., 1976; Baker and Singer, 1988) and delayed relaxation (Meulemans et al., 1990; Brutsaert, 2003). The role of the renin-angiotensin-aldosterone system in cardiac hypertrophy is well characterized and led to the successful implementation of ACE inhibitors and angiotensin receptors blockers in daily clinical practice of heart failure (Weber and Brilla, 1991; Sadoshima and Izumo, 1993; Paul et al., 2006). In the dataset used in this manuscript to select proteins, Angiotensin converting enzyme (ACE) mRNA is upregulated 3.2-fold in ECs after aortic banding (Table 3).

The effects of *Et-1* on cardiac contractility are diverse, but the most reproducible response is a positive inotropic effect (Moravec et al., 1989). Long term activation of the Et-1 pathway induces a hypertrophic response in cardiomyocytes and has been implicated in heart failure soon after its discovery (Drawnel et al., 2013); circulating and tissue levels of Et-1 are increased in patients with heart failure (Lerman et al., 1992; Loffler et al., 1993). However, studies with Et-1 receptor blockers in patients with heart failure have been disappointing (O'connor et al., 2003; Anand et al., 2004). This could be partly explained by the essential role of Et-1 for maintenance of normal cardiac contractility and for the adaptive stress response of cardiac tissue (Hathaway et al., 2015). Furthermore, Et-1 has been shown to have anti-apoptotic properties on cardiomyocytes (Kakita et al., 2001; Ogata et al., 2003; Drawnel et al., 2013). Interestingly, endothelium-specific Et-1 knockout mice show an exaggerated hypertrophic response to aortic banding (Heiden et al., 2014).

## Microvascular endothelial cells secrete proteins that modulate cardiomyocyte function and cardiac remodeling

ECs could be viewed as a single continuous organ of considerable size throughout the organism, instead of an additional cell type present in separate organs. They form an active secretory organ that not only has a major influence on the proteome in blood plasma but also on the proteome in the interstitial space of the capillaries. The secretome of ECs plays an essential role in development and normal physiology of all organs. In the heart, ECs are essential for normal development of the heart through various pathways including Notch and Wnt signaling pathways. The endothelial secretome is crucial in adult myocardium for maintenance of normal myocardial function and adequate response to various hemodynamic stimuli (e.g., pressure overload).

Data from a recently performed micro-array by Moore-Morris et al. show a marked upregulation of various secreted proteins by cardiac microvascular ECs upon chronic pressure overload in mice (Moore-Morris et al., 2014). Upregulated angiocrine proteins with a known cardiac function are shown in Table 3. This list cannot be assumed to be complete, because the micro-array is based on a single sample of sham and TAC operated mice precluding statistical analysis. However, this list is remarkably similar to a list that can be construed by comprehensive review of the literature on the effects of various secreted proteins on cardiac function, hypertrophy and remodeling. The fact that cells were freshly isolated from intact hearts with flow cytometry has two important advantages: the cells that were isolated are pure ECs and gene expression is analyzed on cells in a condition that matches their *in vivo* condition as closely as possible. There are also some drawbacks in using this list of proteins. For example, proteins that regulate cardiac contractility but not cardiac remodeling are perhaps not represented, neither proteins that are regulated by posttranslational modifications instead of transcription. We will use this list of proteins with differential expression between pressure-overloaded and normal hearts as an index list of proteins for further review, but one should keep in mind the advantages and drawbacks discussed.

We confirmed expression of these genes in cardiac microvascular ECs based on publicly available microarray data on various pure cell cultures (GEO Dataset: GDS1402). This microarray experiment contains 16 primary EC cultures, 7 fibroblast cell cultures, and 26 vascular smooth muscle cell cultures. We compared expression of different genes between ECs and fibroblasts and between ECs and vascular smooth muscle cells (Table 3). Table 3 only shows values for expression levels that are significantly different; for a number of proteins—e.g., TSP-4, IGF-1, or BMP-2—expression levels in ECs are comparable to expression in fibroblasts and vascular smooth muscle cells. Some proteins—e.g., IL-1β or prostacyclin synthase—have a higher expression in vascular smooth muscle cells and fibroblasts compared to ECs. Expression of TSP-1, BMP-4, and PGF is markedly higher in ECs compared to fibroblasts or vascular smooth muscle cells. These micro-arrays have been performed on cultured cells and one has to be careful with extrapolating these data to the *in vivo* situation because expression levels may be altered by the artificial cell culture environment and growth factors used in cell culture medium.

We evaluated expression of a panel of angiocrine proteins in micro-array experiments of different heart failure models, including biopsy samples of different forms of cardiomyopathy in humans and different animal models of heart failure (Table 3). Different forms of heart failure are included in this experiment: hypertensive cardiomyopathy, ischemic cardiomyopathy, dilated cardiomyopathy, myocarditis, and obesity induced cardiomyopathy. Most of the angiocrine proteins are up- or down-regulated in one or more of these heart failure models (Table 3), but none of the proteins is significantly altered in all of them. The large variety in underlying pathophysiology of these heart failure models is the main reason for variability in expression levels of angiocrine proteins. We included different models of heart failure, because heart failure is a heterogeneous disease, not only because of different causal factors, but also because of differences in genetic susceptibility, comorbidities, and even differences in a single patient when disease progresses over time. Furthermore, in contrast to the experiment performed by Moore-Morris et al. (first column of Table 3), all these expression data are based on biopsies or tissue samples and therefore are a mixture of different cell types. Although the number of cardiomyocytes and ECs can be expected to remain fairly constant, induction of heart failure will lead to changes in relative abundances of different cell types in the heart and therefore might affect expression levels. Relative changes in cell numbers will be different between different models of heart failure: e.g., fibroblast proliferation is more pronounced in certain models. Another caveat when interpreting Table 3 is the fact that not all genes are included in all micro-arrays, e.g., TSP-3 is only present in a minority of micro-array panels.

Abundance of angiocrine proteins is not only dependent on transcriptional activity, but also on translation, posttranslational modification and secretion. Therefore, we searched literature for mass-spectrometry data on the secretome of ECs. Specific data on cardiac microvascular ECs are not available, but mass spectrometry data have been published on the secretome of HUVECs (Tunica et al., 2009), endothelial progenitor cells (Hemmen et al., 2011), and EA.hy926 ECs (HUVEC hybridoma cell line) (Brioschi et al., 2013; Kwon et al., 2015).

A recent study investigated the *in vitro* response of endothelial responses to endotoxins (Kwon et al., 2015). Although the method used in this study simulates the pathophysiology of sepsis rather than cardiac remodeling, many inflammatory pathways are also activated in cardiac remodeling. Interestingly, endotoxins upregulate secretion of some of the proteins present in our index list; e.g., thrombospondin-1 secretion increases 1.2-fold, follistatin-related protein 1 secretion increases 1.2-fold, and connective tissue growth factor increases 1.8-fold (Kwon et al., 2015; Table 4). In a separate mass-spectrometry study in the same EC line it was shown that atorvastatin decreases protein secretion of thrombospondin-1, thrombospondin-2, and connective tissue growth factor (Brioschi et al., 2013). HMG-CoA reductase inhibitors have been said to have pleiotropic effects on other organ systems besides their cholesterol lowering effects (Mihos et al., 2014). Stimulation or inhibition of specific angiocrine proteins could be part of these pleiotropic effects of statins.

**Table 4:** Expression of angiocrine proteins as determined by mass-spectrometry.

**Gene**	**Protein**	**A**	**B**
		EA.hy926	EA.hy926
		LPS	statin
Thbs1	Thrombospondin 1	1.2	0.3
Fstl1	Follistatin-like 1	1.2	
Ctgf	Connective tissue growth factor	1.8	0.3
Thbs2	Thrombospondin 2		0.4

*(A) Relative expression of angiocrine proteins in EA.hy926 ECs after stimulation with endotoxin (LPS); LC-MS/MS data (Kwon et al., 2015). (B) Relative expression of angiocrine proteins in EA.hy926 ECs after treatment with atorvastatin in vitro; LC-MS/MS data (Brioschi et al., 2013)*.

Fibroblasts are generally considered to be the main source of extracellular matrix proteins, but ECs themselves are an important source of extracellular matrix proteins as well. ECs increase production of extracellular matrix proteins in response to pressure overload (Table 5) and therefore could significantly contribute to formation of extracellular matrix during fibrogenesis. Moreover, ECs also secrete numerous pro-fibrotic factors in response to hemodynamic stress.

**Table 5:** Expression of extracellular matrix proteins by endothelial cells during cardiac overload.

**Gene**	**Protein**	**Fold**
**COLLAGEN**		
Col1a1	collagen, type I, alpha 1	44.7
Col1a2	collagen, type I, alpha 2	59.4
Col3a1	collagen, type III, alpha 1	38.4
Col4a4	collagen, type IV, alpha 4	5.6
Col5a1	collagen, type V, alpha 1	13.3
Col5a2	collagen, type V, alpha 2	20.6
Col6a1	collagen, type VI, alpha 1	16.0
Col6a2	collagen, type VI, alpha 2	7.7
Col6a3	collagen, type VI, alpha 3	16.5
Col8a1	collagen, type VIII, alpha 1	8.1
Col8a2	collagen, type VIII, alpha 2	7.1
Col11a1	collagen, type XI, alpha 1	10.0
Col12a1	collagen, type XII, alpha 1	24.6
Col14a1	collagen, type XIV, alpha 1	20.1
Col15a1	collagen, type XV, alpha 1	2.5
Col16a1	collagen, type XVI, alpha 1	4.6
Col18a1	collagen, type XVIII, alpha 1	7.1
Col27a1	collagen, type XXVII, alpha 1	4.6
**BASEMENT MEMBRANE COMPONENTS**		
Lama2	laminin, alpha 2	6.3
Lamb1	laminin B1	2.3
**MAJOR KNOWN EXTRACELLULAR MATRIX GLYCOPROTEINS**		
Efemp1	epidermal growth factor-containing fibulin-like extracellular matrix protein 1	2.6
Eln	Elastin	2.3
Emid2	EMI domain containing 2	5.3
Emilin1	elastin microfibril interfacer 1	3.3
Emilin2	elastin microfibril interfacer 2	3.5
Fbln1	fibulin 1	7.5
Fbln2	fibulin 2	2.5
Fbln5	fibulin 5	2.9
Fbn1	fibrillin 1	3.7
Fbn2	fibrillin 2	8.3
Fn1	fibronectin 1	3.4
Matn2	matrilin 2	5.4
Mfap4	microfibrillar-associated protein 4	45.0
Mfap5	microfibrillar associated protein 5	36.6
Postn	periostin, osteoblast specific factor	46.7
**PROTEOGLYCAN**		
Aspn	Asporin	7.2
Bgn	Biglycan	8.7
Dcn	Decorin	7.3
Fmod	Fibromodulin	14.0
Gpc6	glypican 6	3.5
Lum	Lumican	21.3
Ogn	Osteoglycin	23.2
Vcan	Versican	40.6
**MMP**		
Mmp14	matrix metallopeptidase 14 (membrane-inserted)	7.8
Mmp2	matrix metallopeptidase 2	26.6
Mmp23	matrix metallopeptidase 23	10.7
Timp1	tissue inhibitor of metalloproteinase 1	60.5
Timp2	tissue inhibitor of metalloproteinase 2	3.5
**EXTRACELLULAR MATRIX PROTEINS OF BONES, CARTILAGE, AND TEETH**		
Dpt	Dermatopontin	6.4
**GROWTH-FACTOR-BINDING-PROTEINS**		
Igfbp4	insulin-like growth factor binding protein 4	3.3
Igfbp5	insulin-like growth factor binding protein 5	2.5
Kcp	kielin/chordin-like protein	4.8
Ltbp2	latent transforming growth factor beta binding protein 2	36.3
**CCN FAMILY PROTEINS**		
Wisp2	WNT1 inducible signaling pathway protein 2	7.6
**ENZYMES**		
Expi	extracellular proteinase inhibitor	3.0
Fuca2	fucosidase, alpha-L- 2, plasma	3.1
Hpse	Heparanase	3.1
Lox	lysyl oxidase	13.3
Loxl1	lysyl oxidase-like 1	24.5
Loxl2	lysyl oxidase-like 2	13.0
Loxl3	lysyl oxidase-like 3	6.8
**OTHER POSSIBLE EXTRACELLULAR MATRIX PROTEINS**		
Aebp1	AE binding protein 1	3.3
Cilp	cartilage intermediate layer protein, nucleotide pyrophosphohydrolase	18.9
Comp	cartilage oligomeric matrix protein	8.5
Crispld2	cysteine-rich secretory protein LCCL domain containing 2	5.3
Cthrc1	collagen triple helix repeat containing 1	23.0
Igsf10	Immunoglobulin superfamily, member 10	5.3
Lgi3	leucine-rich repeat LGI family, member 3	2.1
Pcolce	procollagen C-endopeptidase enhancer protein	4.8
Pcolce2	procollagen C-endopeptidase enhancer 2	6.0
Smoc2	SPARC related modular calcium binding 2	8.7
Spon1	spondin 1, (f-spondin) extracellular matrix protein	2.1
Srpx2	sushi-repeat-containing protein, X-linked 2	21.2
Svep1	sushi, von Willebrand factor type A, EGF and pentraxin domain containing 1	6.8
Tgfbi	transforming growth factor, beta induced	4.5

*Relative expression of different extracellular matrix proteins in cardiac microvascular ECs of mice after thoracic aortic constriction compared to sham operated mice. Based on microarray data of flow cytometry sorted cardiac microvascular ECs (GSE45820) (Moore-Morris et al., 2014)*.

## Endothelium-derived proteins modulating cardiac contractility and cardiac remodeling

In this section, we will discuss endothelium-derived proteins with known effects on cardiac function and/or remodeling. All proteins showed an increased expression in endothelial cells in response to pressure overload (Table 3), and they will be discussed in order of magnitude of this response.

### Interleukin-6

It is well established that inflammatory cytokines including tumor necrosis factor-α, interleukin-1, and interleukin-6 (IL-6) play important roles in early and later stages of cardiac remodeling and heart failure (Paulus, 2000). In heart failure patients, inflammatory cytokines are elevated in the myocardium but also in plasma and have paracrine and endocrine functions (Paulus, 2000). Inflammatory cytokines can be produced by immune cells but also other cell types including ECs. Many excellent reviews cover the role of IL-6 signaling pathways in heart failure and cardiac remodeling (Fischer and Hilfiker-Kleiner, 2007; Fontes J. A. et al., 2015).

The effects of IL-6 on cardiomyocyte contractility have been well documented *in vitro*. IL-6 induces reversible negative inotropic effects on isolated hamster papillary muscles (Finkel et al., 1992), downregulation of SERCA2 in neonatal rat ventricular myocytes (Villegas et al., 2000), reduced expression of cardiac myosin heavy chain isoforms, and a loss of cardiac actin in rat cardiac myocytes (Patten et al., 2001). The negative inotropic effects of IL-6 in isolated cardiomyocytes can be partially explained by stimulation of iNOS expression and NO production (Yu et al., 2003). IL-6 increases NO production through activation of the JAK2/STAT3 pathway (Yu et al., 2003).

**Figure 4:**
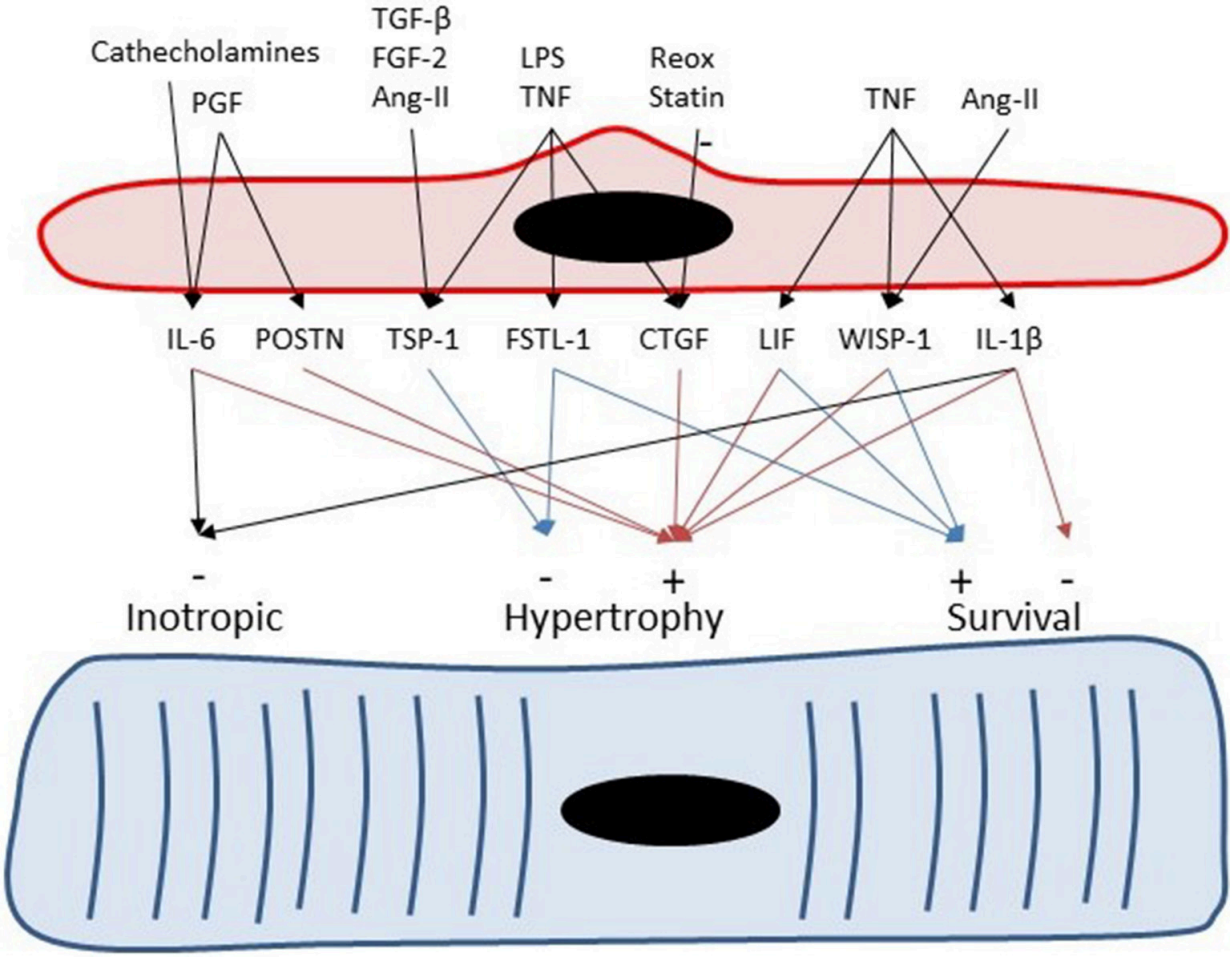
Endothelial cells can serve as paracrine intermediates. Placental growth factor (PGF) induces expression of periostin (Postn) and IL-6 in non-myocytes including cardiac microvascular ECs. Periostin and IL-6 have been implicated to play a role in adaptive hypertrophy induced by PGF (Accornero et al., 2011). Catecholamines induces cardiac hypertrophy partly by induction of endothelium-derived IL-6 (Papay et al., 2013). Transforming growth factor-β, fibroblast growth factor-2 (Frangogiannis et al., 2005), and Ang-II (Fischer et al., 2001) induce TSP-1 expression in CMVECs.

Besides negative inotropic effects, IL-6 also has clear effects on cardiac remodeling. Inhibition of IL-6 in a mouse model of transplant rejection decreases cardiomyocyte hypertrophy and cardiac fibrosis, indicating that IL-6 has pro-hypertrophic and pro-fibrotic properties (Diaz et al., 2009). The pro-hypertrophic effects of IL-6 have been recently confirmed in mice with a genetic deletion of IL-6, which showed attenuation of the hypertrophic response to pressure overload (Zhao et al., 2016) and attenuation of the fibrotic response to Ang-II infusion (González et al., 2015). Moreover, experiments with transgenic mice overexpressing α-adrenergic receptors suggest that part of the hypertrophic response of cardiomyocytes to catecholamines is mediated by endothelium-derived IL-6 (Papay et al., 2013; Figure 4). Furthermore, endothelium-derived IL-6 has also been implicated in the adaptive hypertrophic response to placental growth factor, an endothelial growth factor (Accornero et al., 2011). As discussed in a later section, placental growth factor stimulates EC growth and release of growth factors—including IL-6—from ECs (Accornero and Molkentin, 2011), and thus has indirect trophic effects on myocytes.

Subcutaneous injection of high doses of recombinant IL-6 in wild-type rats induces a dose-dependent dilatation of the left ventricle leading to heart failure within 7 days (Janssen et al., 2005), whereas continuous infusion of a lower doses of IL-6 in rats leads to ventricular hypertrophy and fibrosis (Meléndez et al., 2010). Consistently, administration of an antibody against the IL-6 receptor decreases cardiac remodeling after myocardial infarction in mice (Kobara et al., 2010). These experiments indicate that increased levels of IL-6 have detrimental effects on cardiac remodeling. However, other experiments indicate that IL-6 has protective effects on cardiac biology as well. For instance, IL-6 KO mice show cardiac dilatation, increased accumulation of interstitial collagen, and a decreased capillary density (Banerjee et al., 2009).

### Periostin

Periostin is a secreted protein of 90 kDa, which contains 4 repetitive fasciclin domains and is involved in cell adhesions (Snider et al., 2009). Periostin is involved in normal fibrogenesis but also pathological fibrosis and interacts directly with other extracellular matrix proteins such as fibronectin, tenascin-C, and collagen I/V (Snider et al., 2009). Periostin can serve as a ligand for selected integrins, such as αvβ3, αvβ5, and α4β6, where it can affect the ability of cells to migrate or undergo a epithelial-mesenchymal transformation during pathological disease progression (Conway and Molkentin, 2008). In the adult heart periostin is induced following myocardial infarction, pressure overload, or generalized cardiomyopathy (Conway and Molkentin, 2008; Frangogiannis, 2012). The effects of periostin on cardiomyocyte contractility are unknown, but periostin does play a role in myocardial fibrosis and hypertrophy (Frangogiannis, 2012). It has been shown that periostin knockout mice have reduced fibrosis and hypertrophy following pressure overload, whereas periostin overexpressing transgenic mice spontaneously developed hypertrophy with aging (Oka et al., 2007).

It has been suggested that recombinant periostin had regenerative properties and can induce cardiomyocyte proliferation after myocardial infarction (Kuhn et al., 2007), but these results have been contested by other investigators (Conway and Molkentin, 2008). Therefore, more studies are needed to investigate that regenerative properties of periostin.

### Tenascin-C

Tenascins (Tn) are a family of multimeric extracellular matrix glycoproteins characterized by a N-terminal globular domain and heptad repeats, which facilitate multimerization (Tucker and Chiquet-Ehrismann, 2009). Tenascins play important roles in cell adhesion and motility (Tucker and Chiquet-Ehrismann, 2009). Tn-C is the best characterized tenascin and is highly expressed in tendons and embryonic extracellular matrix (Tucker and Chiquet-Ehrismann, 2009). Tn-C has a wide range of effects on cell adhesion, motility, differentiation, growth control, and extracellular matrix organization via multiple cell surface receptors (Tucker and Chiquet-Ehrismann, 2009). Tn-C is expressed in various ECs including aortic ECs, pulmonary artery ECs, and HUVECs (Golledge et al., 2011; Table 6). Tn-C is secreted by ECs, but also has dynamic effects on ECs by inhibiting cardiac EC spreading and enhancing migration in response to angiogenic growth factors (Ballard et al., 2006). Tn-C has both pro- and antiangiogenic properties (Tucker and Chiquet-Ehrismann, 2009).

**Table 6:** Various effects of endothelial derived proteins.

	**Fibrosis**		**CM contractility**		**CM hypertrophy**		**CM survival**		**CM replication**		**CSC differentiation**	
	**Pro**	**Anti**	**Pos**	**Neg**	**Pro**	**Anti**	**Pro**	**Anti**	**Pro**	**Anti**	**Pro**	**Anti**
Periostin	Oka et al., 2007	Kuhn et al., 2007	?	?	Oka et al., 2007				Kuhn et al., 2007			
DKK-3						Zhang et al., 2014	Bao et al., 2015					
TSP-1	Frangogiannis, 2012					Xia et al., 2011						
TSP-2		Swinnen et al., 2009					Swinnen et al., 2009; van Almen et al., 2011					
TSP-4		Frolova et al., 2012	Cingolani et al., 2011			Palao et al., 2016						
IL-6	Diaz et al., 2009; Meléndez et al., 2010; González et al., 2015			Yu et al., 2003	Diaz et al., 2009; Meléndez et al., 2010; Zhao et al., 2016							
IL-1β	Bujak and Frangogiannis, 2009			Bujak and Frangogiannis, 2009	Bujak and Frangogiannis, 2009			Bujak and Frangogiannis, 2009				
ADM		Kato and Kitamura, 2015	Szokodi et al., 1998; Ihara et al., 2000	Ikenouchi et al., 1997; Mukherjee et al., 2002		Kato and Kitamura, 2015						
LIF					Kodama et al., 1997		Zouein et al., 2013				Zouein et al., 2013	
Wisp-1	Colston et al., 2007				Colston et al., 2007		Venkatesan et al., 2010					
Midkine					Netsu et al., 2014		Kadomatsu et al., 2014					
BMP-2		Wang et al., 2012	Ghosh-Choudhury et al., 2003			Lu et al., 2014	Lu et al., 2014				DeGeorge et al., 2008; Kim et al., 2011; Wan et al., 2011	
BMP-4	Sun et al., 2013				Lu et al., 2014			Lu et al., 2014			Hosseinkhani et al., 2007	Taha et al., 2007
Apelin		Pchejetski et al., 2012; Zhang et al., 2016; Zhong et al., 2016	Szokodi et al., 2002; Ashley et al., 2005									
PGF			Accornero et al., 2011									
FSTL-1	Dong et al., 2015; Maruyama et al., 2016					Shimano et al., 2011	Oshima et al., 2008; Ogura et al., 2012		Wei et al., 2015			
CTGF					Hayata et al., 2008							
IGF-1			Ren et al., 1999		Ren et al., 1999		Ren et al., 1999		Ren et al., 1999		Suleiman et al., 2007	
Tenascin	Imanaka-Yoshida, 2012											

Tn-C is almost absent in normal adult myocardium, but reappears during cardiac remodeling in response to pathologic insults, such as acute myocardial infarction, myocarditis, ischemia-reperfusion injury, and hypertensive cardiac fibrosis (Imanaka-Yoshida, 2012). Studies on direct effects of Tn-C on cardiomyocyte hypertrophy are missing, but Tn-C plays an important role during cardiac remodeling by loosening cell adhesion, upregulating MMPs, and enhancing inflammatory responses (Imanaka-Yoshida, 2012). These effects of Tn-C help cell rearrangement and allow myofibroblasts and capillary vessels to spread into the restoring tissue, but these might also cause tissue vulnerability, resulting in ventricular dilatation (Imanaka-Yoshida, 2012). Consistently, deletion of TN-C significantly reduces ventricular remodeling and improves cardiac function after coronary artery ligation in mice (Nishioka et al., 2010).

### Thrombospodins

Expression of thrombospondin-4 (TSP-4) has been documented in human coronary artery ECs and smooth muscle cells (Stenina et al., 2003). Pressure overload leads to an increase in TSP-1 and TSP-4 expression in cardiac tissue (Mustonen et al., 2008; Xia et al., 2011), which confirms the micro-array dataset of Table 3. More specifically, immunostaining localized TSP-4 to ECs in hypertrophied hearts of spontaneously hypertensive rats (Mustonen et al., 2008). Upregulation of TSP-4 expression by increased loading conditions has also been shown in models of vasopressin infusion or myocardial infarction (Dawson et al., 2013). Therefore, it has been suggested that TSP-4 is an endothelial specific marker of cardiac overload (Mustonen et al., 2008). TSP-1 also shows load-dependent expression in ECs (Dawson et al., 2013), but compared to TSP-4 also shows a more widespread expression in different cell types (Frangogiannis et al., 2005). Expression of TSP-1 in ECs is also regulated by transforming growth factor-β, fibroblast growth factor-2 (Frangogiannis et al., 2005), and Ang-II (Chua et al., 1997; Fischer et al., 2001) (Figure 4).

TSP-4 serves as an endothelium-derived mechano-signaling molecule with a central role in adaptive contractile responses to acute stress, and appears to play a crucial role in the transition to chronic cardiac dilatation (Cingolani et al., 2011). Hearts of TSP-4 KO mice failed to acutely augment contractility or activate stretch-response pathways on exposure to acute pressure overload (Cingolani et al., 2011). In these complete KO mice, TSP-4 was deleted in all cells. Isolated cardiac trabeculae of TSP-4 KO mice failed to enhance contractility and cellular calcium increase after stretch, in contrast to wild-type trabeculae (Cingolani et al., 2011). However, the contractility response could be restored in TSP-4 KO cardiac trabeculae incubated with recombinant TSP-4 (Cingolani et al., 2011). Interestingly, when TSP-4 KO myocytes were isolated, they responded normally to stretch, indicating that TSP-4 secreted by other cells—e.g., microvascular ECs—is crucial for a normal response to stretch.

TSP-1 KO mice display an increased hypertrophic response to pressure overload (Xia et al., 2011). Similarly, TSP-4 KO mice also display an increased hypertrophic response (Frolova et al., 2012; Palao et al., 2016). Little is known on the role of TSP-2 in cardiomyocyte hypertrophy, but TSP-2 has been shown to be protective against doxorubicin induced cardiomyocyte toxicity (van Almen et al., 2011). Furthermore, TSP-2 also protects against age-related dilated cardiomyopathy in mice (Swinnen et al., 2009). In summary, TSP-1,−2,−3, and−4 are upregulated in ECs during pressure overload, but most data in the literature focus on TSP-1 and−4, that both seem to serve as endothelium-derived anti-hypertrophic factors.

Besides having anti-hypertrophic properties, TSP-1 is an important activator of transforming growth factor-β signaling. Latency-associated peptide is a peptide that forms a complex with transforming growth factor-β and in this state inactivates transforming growth factor-β. Binding of TSP-1 to latency-associated peptide leads to a conformational change leading to release and activation of transforming growth factor-β (Frangogiannis, 2012). As stated before, TSP-1 expression is increased by pressure overload and plays an important role in the fibrotic response of myocardial tissue. TSP-1 null mice have a low collagen content in the heart with increased infiltration of dysfunctional fibroblasts (Xia et al., 2011). Hearts of TSP-1 null mice are prone to chamber dilatation in response to pressure overload because of loss of extracellular matrix integrity. In contrast to TSP-1 KO mice, TSP-4 KO mice display an increased deposition of extracellular matrix in response to pressure overload (Frolova et al., 2012), indicating that TSP-4 serves as an endothelium-derived suppressor of exaggerated fibrosis. It has also been shown that TSP-2 has anti-fibrotic effects in age-related cardiac dilatation (Swinnen et al., 2009).

### Follistatin-like 1

Follistatin-like 1 (FSTL-1) is an extracellular glycoprotein with limited homology to the follistatin family of proteins (Ouchi et al., 2008). It has been reported that FSTL-1 is secreted by skeletal myocytes (Ouchi et al., 2008), myocardial tissue after myocardial infarction (Oshima et al., 2008), and cardiomyocytes and nonmyocytes (Shimano et al., 2011). Expression of FSTL-1 in ECs has been confirmed by immunohistochemistry in synovial ECs (Li et al., 2011) and cardiac microvascular ECs (Shimano et al., 2011). Numerous studies indicate that FSTL-1 has cardioprotective properties during cardiac remodeling. FSTL-1 has anti-apoptotic effects on cardiomyocytes by stimulation of the AKT pathway (Oshima et al., 2008). Furthermore, FSTL-1 shows anti-hypertrophic properties in murine animal models (Shimano et al., 2011; Tanaka et al., 2016). Data from small and large animal models also indicate that FSTL-1 has protective effects on ischemia/reperfusion injury (Ogura et al., 2012). Moreover, data indicate that FSTL-1 is upregulated after myocardial infarction and prevents cardiac rupture by activating cardiac fibroblasts (Maruyama et al., 2016). Consistently, FSTL-1 also has pro-fibrotic effects in models of lung fibrosis (Dong et al., 2015).

### Frizzled-related protein 3

Frizzled-related protein 3 (FRP-3) is a member of the Wnt signaling pathway and plays important roles during cardiac embryogenesis. Expression of FRP-3 in ECs has also been confirmed by immunohistological staining in reproductive tissues (Partl et al., 2014). Currently, there are no known effects of FRP-3 on cardiac contractility, but it has been shown that FRP-3 expression is increased in failing human myocardium, with a decline following LV assist device therapy (Askevold et al., 2014), indicating that FRP-3 is related to heart failure progression, and its secretion dependent on ventricular wall strain. It has also been shown that FRP-3 mRNA levels are increased in left ventricles of mice post-myocardial infarction (Askevold et al., 2014). Like other members of the Wnt signaling pathway, evidence indicates that FRP-3 plays a role in cardiac remodeling, but direct effects on cardiomyocytes or fibroblasts have not been examined yet.

### Insulin-like growth factor-1

Insulin-like growth factor-1 (IGF-1) is a protein with growth promoting actions on multiple tissues including the myocardium. IGF-1 is part of the growth hormone /IGF-1 pathway and promotes cell survival via the phosphatidylinositol 3 kinase (PI3K)/Akt signaling pathway (Figure 4; Michele et al., 2013). Expression of IGF-1 has also been confirmed in brain microvascular ECs (Wang et al., 2013), endothelial progenitor cells (Urbich et al., 2005), and adipose tissue microvascular ECs (Kern et al., 1989).

It has been well-established that IGF-1 plays important roles in physiological and pathological cardiac remodeling and heart failure (Ellison et al., 2012). IGF-1 promotes cardiac growth and improves cardiac contractility and ejection fraction (Ren et al., 1999). IGF-1 also improves cardiac function after myocardial infarction by promoting tissue remodeling (Ren et al., 1999). We refer the reader to many excellent reviews on the role of IGF-1 in cardiac remodeling (Ren et al., 1999; Opgaard and Wang, 2005; Michele et al., 2013).

### Connective tissue growth factor

Connective tissue growth factor (CTGF) is a protein that plays an essential role in skeletal development and extracellular matrix production (Accornero et al., 2015). CTGF plays crucial roles in fibrotic responses, for instance in models of bleomycin-induced skin fibrosis (Liu et al., 2010). CTGF binds with various proteoglycans and integrins, but a specific cell surface receptor for CTGF has not been identified, so it is currently unclear how CTGF modifies cellular responses to injury. Although CTGF expression is strongly induced during cardiac remodeling, its role in cardiac remodeling remains controversial. Recent studies using transgenic mice suggest that CTGF is not involved in cardiac remodeling, hypertrophy, or fibrosis at baseline, nor with aging, after pressure overload, or with neuroendocrine agonist stimulation (Accornero et al., 2015; Fontes M. S. et al., 2015). However, *in vitro* data indicate that CTGF induces hypertrophy in cardiomyocytes (Hayata et al., 2008).

### Dickkopf-3 (DKK-3)

Dickkopf (DKK) proteins are secreted regulators of the Wnt signaling pathway which include DDK-1 to DKK-4 (Krupnik et al., 1999; Niehrs, 2006). DKK-3 plays a role in embryological development of various organs including the heart, bone, the neural epithelium, and limb buds (Niehrs, 2006). DKK-3 is also expressed in various adult tissues and acts as a tumor suppressor of different malignancies (Veeck and Dahl, 2012). Expression of DKK-3 in tumor tissues is mostly located in microvascular ECs (Untergasser et al., 2008; Fong et al., 2009). DKK-3 is expressed in the adult heart (Krupnik et al., 1999), more specifically in microvascular ECs (Fong et al., 2009; Tunica et al., 2009; Zhang et al., 2014).

DKK-3 has been identified as a protein playing a role in cardiac remodeling. Genomic studies have shown that DKK3 is negatively correlated with myocardial mass in rat models of hypertrophy (Cerutti et al., 2006). Furthermore, it has been shown that overexpression of DKK-3 protects against aortic banding induced hypertrophy whereas knockout of DKK-3 aggravates the hypertrophic response (Zhang et al., 2014). It has also been shown that DKK-3 has protective effects in models of dilated cardiomyopathy (Lu et al., 2016) and myocardial infarction (Bao et al., 2015).

### Bone morphogenetic protein-2 and -4

Bone morphogenetic protein-2 (BMP-2) and bone morphogenetic protein-4 (BMP-4) play important roles during heart development in vertebrates and are members of the transforming growth factor-β superfamily (Balligand et al., 2009). The role of BMP-2 in regeneration of the adult heart is incompletely defined, but BMP-2 is frequently used to stimulate differentiation of stem cells for cardiac regeneration therapies (DeGeorge et al., 2008; Kim et al., 2011; Wan et al., 2011). Similarly, BMP-4 has been shown to induce differentiation of embryonic stem cells (Hosseinkhani et al., 2007) and induced pluripotent stem cells into cardiomyocytes (Ren et al., 2011). However, BMP-4 has also been reported to have inhibiting properties on cardiomyocyte differentiation (Taha et al., 2007). It has been shown that cyclic stretch of ECs *in vitro* induces expression of BMP-2 (Balligand et al., 2009) and that BMP-2 has positive inotropic effects on isolated adult cardiomyocytes by activation of PI3K (Ghosh-Choudhury et al., 2003).

Recent evidence indicates that BMP-4 as well as BMP-2 also play key roles in cardiac remodeling. Whereas BMP-2 has anti-hypertrophic effects on adult cardiomyocytes, BMP-4 has pro-hypertrophic and pro-apoptotic effects on cardiomyocytes (Sun et al., 2013; Lu et al., 2014). Moreover, BMP-4 also has pro-fibrotic effects (Sun et al., 2013) whereas BMP-2 has anti-fibrotic effects (Wang et al., 2012).

Moreover, BMP-2 promotes angiogenesis in various tumors (Langenfeld and Langenfeld, 2004) and during osteogenesis (Carano and Filvaroff, 2003). BMP-4 has also potent pro-angiogenic properties and induces capillary sprouting (Zhou et al., 2007).

### Apelin

Apelin is the ligand for the previously orphaned G protein–coupled receptor APJ (Japp et al., 2010). Apelin is expressed throughout the organism with particularly high levels in vascular endothelium (Chandrasekaran et al., 2008). APJ receptors are present on many different cell types including ECs, cardiomyocytes, and vascular smooth muscle cells (Japp et al., 2010). Apelin exerts major effects on both vascular tone and cardiac contractility. Both in isolated rat hearts and *in vivo*, apelin is a positive inotropic substance (Szokodi et al., 2002; Ashley et al., 2005). Together with Et-1 and adrenomedullin, apelin is among the most potent endogenous inotropic substances on a molar base (Szokodi et al., 2002). Apelin exerts its inotropic action by increasing the availability of intracellular calcium rather than enhancing myofilament calcium sensitivity (Chandrasekaran et al., 2008). In the failing heart, this increase in contractility is even more pronounced (Chandrasekaran et al., 2008). Apelin not only increases inotropy, but also decreases left ventricular pre- and afterload by its pronounced vasodilatory effects (Ashley et al., 2005). Therefore, apelin seems to be a beneficial endothelium-derived protein that increases inotropy and decreases cardiac loading when the myocardium is confronted with pressure overload. Interestingly, unlike most other inotropic agents, apelin does not induce cardiomyocyte hypertrophy (Chandrasekaran et al., 2008).

It has recently been shown that apelin also has anti-fibrotic effects in models of pressure overload (Pchejetski et al., 2012; Zhong et al., 2016) and myocardial infarction (Zhang et al., 2016) with direct inhibitory effects on fibroblasts (Pchejetski et al., 2012; Zhong et al., 2016).

### Interleukin-1β

Interleukin-1β (IL-1β) is an inflammatory cytokine which is expressed in multiple tissues and by multiple cell types including ECs. In experimental models of pressure overload and cardiac hypertrophy, IL-1β expression is upregulated in the hypertrophied heart, predominantly localized in ECs and interstitial macrophages (Bujak and Frangogiannis, 2009). Similar to IL-6, IL-1β also has a negative inotropic effect on cardiomyocytes (Bujak and Frangogiannis, 2009). This negative inotropic effect is mediated through NO-dependent and NO-independent pathways (Bujak and Frangogiannis, 2009). Furthermore, IL-1β inhibits the β-adrenergic agonist-mediated increase in cAMP and cardiomyocyte contractility and IL-1β is an essential mediator in sepsis-induced contractile dysfunction (Bujak and Frangogiannis, 2009).

Extensive evidence suggests that IL-1 has pro-hypertrophic and pro-apoptotic effects on cardiomyocytes (Bujak and Frangogiannis, 2009). IL-1β induces cardiomyocyte apoptosis by activation of Bak and Bcl-xL through pathways involving NO (Bujak and Frangogiannis, 2009). Furthermore, IL-1β induces cardiomyocyte hypertrophy, upregulates atrial natriuretic factor (ANF) and suppresses expression of calcium regulatory genes (Bujak and Frangogiannis, 2009). Furthermore, IL-1β has well known pro-inflammatory properties. In IL-1-receptor KO hearts, collagen deposition was markedly decreased, in both the healing scar and the peri-infarct area (Bujak and Frangogiannis, 2009). IL-1β directly enhances fibrosis by upregulating expression of Ang-II receptors on cardiac fibroblasts and by stimulating fibroblast migration (Bujak and Frangogiannis, 2009). Beyond its pro-inflammatory and fibrogenic properties, IL-1 also promotes extracellular matrix remodeling by enhancing matrix metalloproteinase expression (Bujak and Frangogiannis, 2009).

### Placental growth factor

Placental growth factor (PGF) is secreted by cardiomyocytes (Accornero and Molkentin, 2011) but also by cardiac microvascular ECs in which expression is upregulated by chronic pressure overload (Table 3) (Accornero and Molkentin, 2011; Moore-Morris et al., 2014). PGF is part of the vascular endothelium growth factor superfamily and binds to the VEGF-1 receptor which is expressed by ECs (Accornero et al., 2011). PGF has a limited role in normal cardiac homeostasis, but has been shown to be crucial in adaptive angiogenic responses (Accornero and Molkentin, 2011). Because CMVECs are at the same time both secretor and receptor cells for PGF, PGF might be part of an autocrine endothelial signaling system.

Deletion or overexpression of PGF does not alter cardiac function or morphology at baseline, but PGF is an essential component of the hypertrophic response to pathological stimuli such as pressure overload (Accornero et al., 2011). In contrast to wild-type mice, PGF KO mice do not form additional capillaries in response to aortic banding and rapidly develop heart failure (Accornero et al., 2011), whereas mice overexpressing PGF show an increased angiogenic response. PGF expression increases in response to hypertrophic stimuli (Accornero and Molkentin, 2011) and stimulates EC growth but also secretion of growth factors from ECs and fibroblasts, including IL-6 and periostin (Accornero and Molkentin, 2011). These growth factors stimulate cardiomyocyte cell growth. Based on these data, it has been suggested that PGF is a stress-response factor that suppresses disease in the heart by maintaining capillary/vessel density as well as providing protective trophic effects to cardiomyocytes (Accornero and Molkentin, 2011).

### Leukemia inhibitory factor

Leukemia inhibitory factor (LIF) is a member of the IL-6 family of cytokines and induces hypertrophy in cardiomyocytes by activation of the LIF receptor and gp130, which functions as a co-receptor (Kodama et al., 1997). The hypertrophic effects of LIF are mediated by the JAK-STAT signaling pathway (Kodama et al., 1997). Most of these pro-hypertrophic effects have been demonstrated in isolated cardiomyocytes, but little is known on the role of LIF in pathophysiology of cardiac remodeling or heart failure (Zouein et al., 2013). However, a large body of evidence exists on the protective effects of the JAK-STAT pathway during acute cardiac stress (Zouein et al., 2013) and it has been shown that LIF protects against ischemia-reperfusion injury (Zouein et al., 2013).

LIF may play a role in cardiac regeneration as it has been shown to contribute to homing of bone marrow-derived progenitors, as well as differentiation of resident cardiac stem cells into ECs (Zouein et al., 2013). LIF not only protected against cardiomyocyte death in a mouse myocardial infarction model, but enhanced neovascularization, and induced homing of bone marrow cells to the heart and their differentiation into cardiac myocytes (Zouein et al., 2013).

### Wnt1-induced secreted protein-1 (WISP-1)

Wnt1-induced secreted protein-1 (WISP-1) is a member of the cysteine-rich 61, CTGF, and nephroblastoma overexpressed (CCN) family of growth factors (Colston et al., 2007). WISP-1 expression is upregulated during cardiac remodeling and expression is stimulated by tumor necrosis factor (Venkatachalam et al., 2009). It has been shown that WISP-1 induces cardiomyocyte hypertrophy *in vitro* by activation of Akt (Colston et al., 2007) and protects against doxorubicin-induced cardiomyocyte death (Venkatesan et al., 2010). It has also been shown that WISP-1 expression is induced by Ang-II stimulation and that WISP-1 is an important mediator of Ang-II induced cardiomyocyte hypertrophy (Shanmugam et al., 2011). WISP-1 also has pro-fibrotic effects by inducing fibroblast proliferation (Colston et al., 2007). Data on the role of WISP-1 are mostly limited to *in vitro* experiments and the role of WISP-1 in cardiac remodeling *in vivo* is largely unexplored.

### Midkine

Midkine is an heparin-binding growth factor that binds to different receptors forming a multireceptor complex (Yamazaki et al., 1998). Midkine protects the heart from ischemia/reperfusion injury and infarction via its anti-apoptotic effects (Kadomatsu et al., 2014). Furthermore, midkine promotes EC proliferation, leading to angiogenesis and it also enhances inflammatory cell infiltration into lesions (Kadomatsu et al., 2014). The pro-angiogenic effects of midkine have been implicated to be the major reason why midkine protects against cardiac remodeling after myocardial infarction (Takenaka et al., 2009). The downside of midkine being a strong pro-angiogenic protein is that it has growth stimulating effects on different tumors. Although midkine has protective effects after myocardial infarction, it has also been shown to increase cardiac hypertrophy during pressure overload (Netsu et al., 2014).

### Adrenomedullin

Adrenomedullin (ADM) is a 52-amino acid protein which belongs to the calcitonin gene-related peptide family. ADM is mainly produced by ECs and vascular smooth muscle cells in different organs (Krzeminski, 2016). ADM is a potent vasodilator that reduces systemic and pulmonary vascular resistance, induces renal vasodilation, and increases glomerular blood flow and filtration rate (Krzeminski, 2016). Research indicates that ADM has positive inotropic effects which involve the activation of adenylyl cyclase and cyclic AMP production in cardiomyocytes (Szokodi et al., 1998; Ihara et al., 2000). It has also been shown that ADM enhances cardiac contractility via mechanisms involving facilitation of Ca^2+^ release (Krzeminski, 2016). However, the effect of ADM on myocardial contractility is controversial because some authors claim it to have a negative inotropic effect mediated by the NO-cGMP pathway or to have no effect on myocardial contractility (Ikenouchi et al., 1997). Another report shows that ADM has negative inotropic effects on human isolated ventricular myocytes (Mukherjee et al., 2002). These discrepancies could partly be explained by interspecies variability in contractile responses.

ADM also has anti-hypertrophic effects and anti-fibrotic effects, thus protects the heart during cardiac remodeling (Kato and Kitamura, 2015). Moreover, ADM also has pro-angiogenic effects in different tissues (Kato and Kitamura, 2015). Taken together, current evidence indicates that ADM is beneficial in a number of cardiovascular diseases because it has protective effects on cardiac remodeling.

## Angiocrine proteins as biomarkers for cardiac disease

ECs are the only cells in the myocardium that are in direct contact with circulating blood. Therefore, proteins secreted by cardiac ECs are more likely to reach the circulation—and will do so in higher concentrations—than proteins from other cell types in the heart. Therefore, specific proteins secreted by ECs could serve as biomarkers of heart failure or cardiac remodeling.

All the proteins discussed in the current paper have been shown to be upregulated in an animal model of pressure overload (Moore-Morris et al., 2014). Some of the proteins discussed in this paper also have been shown to have increased circulation plasma levels in patients with heart failure. For instance, a large body of evidence indicates that circulating levels of IL-6 are increased in patients with heart failure and provide important prognostic information (Wollert and Drexler, 2001). Current evidence on circulating proteins in different forms of heart failure is presented in Table 7. Endothelium-derived proteins can be up- or down-regulated in different forms of heart failure. For instance circulating periostin levels are decreased after myocardial infarction (Cheng et al., 2012), but are increased in patients with dilated cardiomyopathy (Norum et al., 2017).

**Table 7:** Circulating endothelial-derived proteins as biomarkers for cardiac disease.

	**HFrEF**	**HFpEF**	**AMI**
Periostin	Norum et al., 2017		Cheng et al., 2012
TSP-2	Hanatani et al., 2014	Kimura et al., 2016	
IL-6	Roig et al.; Tsutamoto et al., 1998	Wu et al., 2011	Miyao et al., 1993
IL-1β			Hasdai et al., 1996
ADM	Jougasaki et al., 1995; Nishikimi et al., 1995	Yu et al., 2001	Kobayashi et al., 1996
Midkine	Kitahara et al., 2010		
Apelin			Liu et al., 2015
PGF	Nakamura et al., 2009		Bui et al., 2012
FSTL-1		Tanaka et al., 2016	
CTGF	Koitabashi et al., 2008	Wu et al., 2014	
IGF-1	Al-Obaidi et al., 2001		Yamaguchi et al., 2008
Tenascin	Terasaki et al., 2007		Sato et al., 2012
FRP-3	Askevold et al., 2014		

## Conclusions

**Figure 5:**
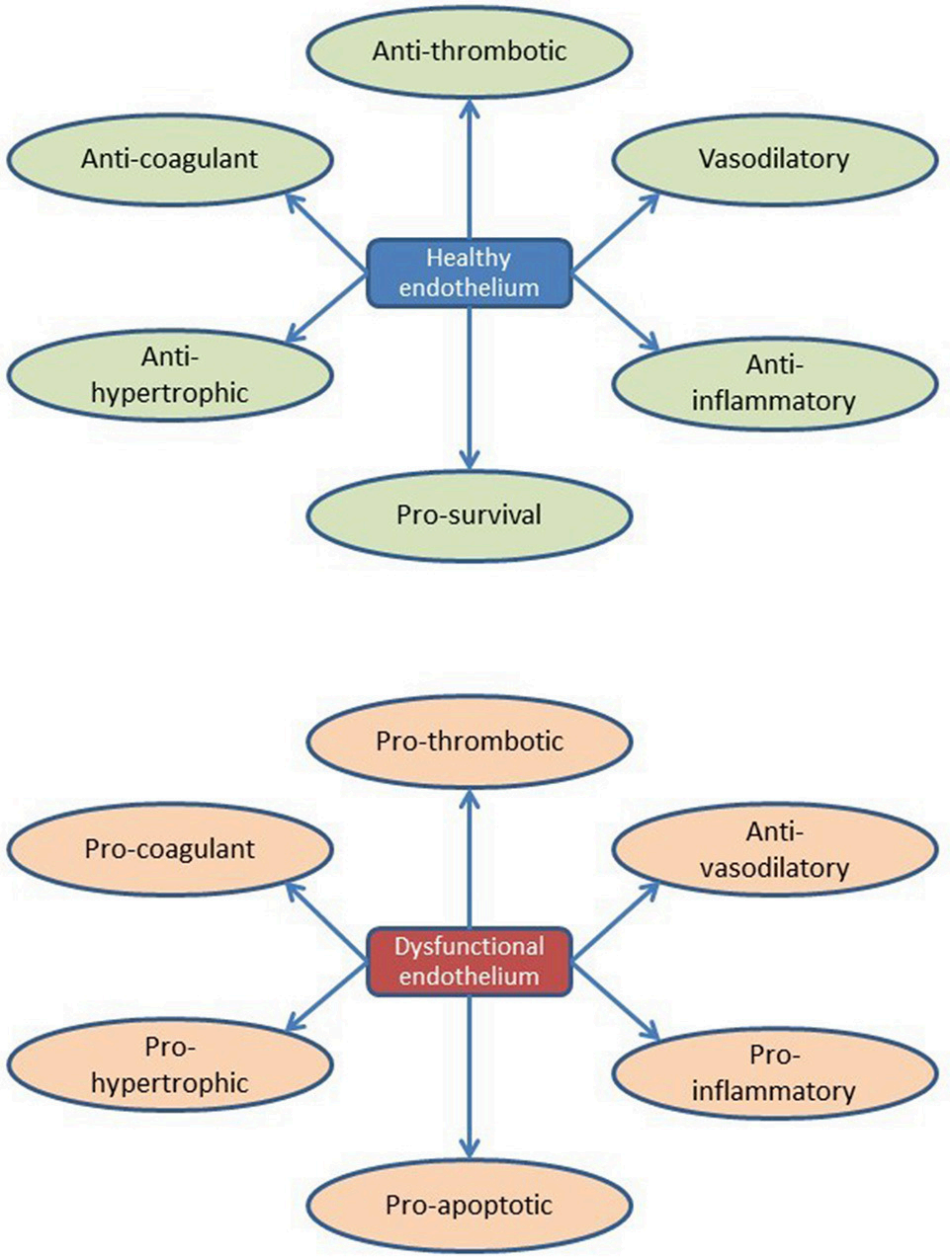
Overview of endothelial function and dysfunction during cardiac remodeling.

By listing currently known secreted endothelial-derived proteins and summarizing their effects on cardiac function or remodeling, an extended view on the (cardiac) endothelium as an (intrinsic) modulatory component of cardiac function emerges. It illustrates the diversity of paracrine pathways through which the endothelium affects the multiple functions and adaptive responses of the heart, which obviously is more complicated than secretion of nitric oxide. Accordingly, there is little doubt that a state of “endothelial activation” or “endothelial dysfunction” has a larger impact on cardiac function and heart failure progression than currently anticipated (and may diverge from the traditional NO-derived views, too often related to the pathophysiology of atherosclerosis; Figure 5).

Given the complexity of the cross-talk between ECs and cardiomyocytes, one may wonder what is missing in our current understanding: (1) For many proteins, stimuli of synthesis and secretion from ECs are incompletely defined. Figure 2 gives a non-exhaustive summary of known stimuli, but these might differ between different proteins. (2) Also, the target cells of endothelium-derived proteins are incompletely characterized. In the current review, we focused on cardiomyocytes, but most proteins have an effect on multiple cell types. (3) We described the actions of different secreted proteins separately, but in reality actions of different proteins are not isolated from one another but enhance or oppose each other. Classically, cardiovascular experiments study the effect of one actor (e.g., a secreted protein) on one target response in a particular cell type (e.g., cardiomyocyte hypertrophy) at one level of complexity (e.g., cellular level). In these “one-dimensional” experiments, however, a lot of information is lost because only a single response is analyzed at a single level of complexity and—at the same time—data on interactions between different pathways and at different levels of complexity are not recorded. A more integrated approach will be necessary to study interdependency and synergy of different pathways. Ultimately, unraveling of paracrine signaling networks will be necessary to fully understand cardiac biology. (4) Drugability of the different paracrine pathways is still largely unexplored, with some notable exceptions such as NO, inflammatory factors, or neuregulin-1. (Segers and Lee, 2010, 2011; De Keulenaer et al., 2017). (5) In the future, endothelial function and dysfunction might have to be redefined as we learn more about other factors secreted by ECs. Currently, definition and evaluation of endothelial function is mostly based on secretion of NO and vasodilatory responses.

## Author contributions

VS: designed and wrote the manuscript; DB and GD: critically revised the manuscript.

### Conflict of interest statement

The authors declare that the research was conducted in the absence of any commercial or financial relationships that could be construed as a potential conflict of interest.
